# Study of the antibacterial effects of the starch-based zinc oxide nanoparticles on methicillin resistance *Staphylococcus aureus* isolates from different clinical specimens of patients from Basrah, Iraq

**DOI:** 10.3934/microbiol.2023006

**Published:** 2023-02-15

**Authors:** Reham M. Al-Mosawi, Hanadi Abdulqadar Jasim, Athir Haddad

**Affiliations:** 1 Department of Microbiology, Dentistry College of Basic Science, University of Basrah, Basrah, Iraq; 2 Department of Microbiology, College of Medicine, University of Basrah, Bashrah, Iraq; 3 Chemistry Department, College of Science, University of Basrah, Basrah, Iraq

**Keywords:** antibiotic resistance, antimicrobial, Iraq, MRSA, ZnO-NPs

## Abstract

This study aimed to assess the efficacy of starch-based zinc oxide nanoparticles (ZnO-NPs) against methicillin-resistant *Staphylococcus aureus* (MRSA) isolates from clinical specimens in Basrah, Iraq. In this cross-sectional study, 61 MRSA were collected from different clinical specimens of patients in Basrah city, Iraq. MRSA isolates were identified using standard microbiology tests, cefoxitin disc diffusion and oxacillin salt agar. ZnO-NPs were synthesized in three different concentrations (0.1 M, 0.05 M, 0.02 M) by the chemical method using starch as the stabilizer. Starch-based ZnO-NPs were characterized using ultraviolet–visible spectroscopy (UV-Vis), X-ray diffraction (XRD), field emission scanning electron microscopy (FE-SEM), energy dispersive X-ray spectroscopy (EDS), and transmission electron microscopy (TEM). The antibacterial effects of particles were investigated by the disc diffusion method. The minimum inhibitory concentration (MIC) and minimum bactericidal concentration (MBC) of the most effective starch-based ZnO-NPs were determined using a broth microdilution assay. The UV-Vis of all concentrations of starch-based ZnO-NPs exhibited a strong absorption band at 360 nm which was characteristic of the ZnO-NPs. XRD assay confirmed the representative hexagonal wurtzite phase of the starch-based ZnO-NPs, and their purity and high crystallinity. The spherical shape with a diameter of 21.56 ± 3.42 and 22.87 ± 3.91 was revealed for the particles by FE-SEM and TEM, respectively. EDS analysis confirmed the presence of zinc (Zn) (61.4 ± 0.54%) and oxygen (O) (36 ± 0.14%). The 0.1 M concentration had the highest antibacterial effects (mean ± SD of inhibition zone = 17.62 ± 2.65 mm) followed by the 0.05 M concentration (16.03 ± 2.24 mm) and the 0.02 M concentration (12.7 ± 2.57 mm). The MIC and the MBC of the 0.1 M concentration were in the range of 25–50 µg/mL and 50–100 µg/mL, respectively. Infections caused by MRSA can be treated with biopolymer-based ZnO-NPs as effective antimicrobials.

## 1. Introduction

*Staphylococcus aureus* is a Gram-positive bacterium and commensal microorganism that colonizes about 30% of the anterior nares of human individuals [1]. This bacterium plays a considerable role in causing both nosocomial and community-acquired infections including skin infections, endocarditis, osteomyelitis, bacteremia, necrotizing pneumonia, toxic shock syndrome, infections associated with foreign bodies, post-operative surgical infections, and food poisoning [2]–[4].

During the past few decades, treating infections caused by *S. aureus* has become challenging due to the emergence of multidrug-resistant, particularly methicillin-resistant *Staphylococcus aureus* (MRSA) [5]. The antimicrobial resistance including methicillin resistance in the MRSA strains is correlated with the acquisition of a mobile genetic element called staphylococcal chromosomal cassette *mec* (SCC*mec*), which harbors both the *mecA* or *mecC* genes, which are responsible for the production of proteins with low binding affinity for beta-lactam antibiotics such as PBP2a [5],[6]. Today, the emergence of MRSA strains resistant to linezolid, vancomycin, and daptomycin has been reported [7].

Since antimicrobial resistance has emerged, spread, and endured in MRSA strains, it has become imperative to develop new and effective alternatives to traditional antibiotics to treat the infections caused by these pathogens [5]. In this regard, nanotechnology can be used to develop antimicrobial nanomaterials with more effective properties compared with traditional antibiotics [5]. It is largely due to their nanoscale size and distinct structures that nanomaterials including inorganic nanoparticles have demonstrated a novel and developed biological functions [8]. Recent studies have found that zinc oxide nanoparticles (ZnO-NPs) possess safe and stable properties that make them one of the ideal antibacterial agents [5]–[8]. There has been speculation that the antimicrobial activity of ZnO-NPs comes from a free radical formation on the surface of metal oxide, which destroys bacterial cell walls and inhibits their growth [8]. Since the experiments conducted in this field are rarely seen in Iraq, this study aimed to assess the efficacy of starch-based synthesized ZnO-NPs against MRSA isolates collected from clinical specimens in Basrah city, Iraq.

## 2. Materials and methods

### 2.1. Ethics statement

This study was approved by the University of Basrah, Basrah, Iraq according to the Declaration of Helsinki. All methods were performed in accordance with the relevant guidelines and regulations of the University of Basrah, Basrah, Iraq. The clinical samples were collected as routine clinical care for referred and admitted patients and not for this study. Hence, the written informed consent was waived by the University of Basrah, Basrah, Iraq.

### 2.2. Study design and sample collection

A total of 150 clinical specimens including wound swabs, sputum, throat swabs, nasal swabs, pus, and urine were collected from patients suffering of urinary tract infection (UTI), wound infection, and upper respiratory tract infection. These patients attended the outpatients and inpatients clinics of Alsadr Teaching Hospital and Al-Shefa General Hospital, Basrah, Iraq for a seven-month period from 1 January to 30 July 2022. All samples were collected in sterile conditions with sterile containers and transmitted to the microbiology laboratory of the College of Medicine, University of Basrah for isolation and identification of MRSA isolates.

### 2.3. Bacterial isolation and identification

Each clinical sample was directly inoculated into plates of mannitol salt agar (MSA, Merck, Darmstadt, Germany) and sheep blood agar (SBA, Merck, Darmstadt, Germany) and incubated at 37 °C for 24–48 h. Then, all colonies from primary cultures were identified depending on the morphological features in culture media as beta hemolytic on blood agar and fermentation of the mannitol sugar on MSA. In addition, a panel of standard microbiology and biochemical tests including Gram staining, catalase, DNase, slide and tube coagulase were performed to confirm the *S. aureus* isolates [9]–[11]. *S. aureus* ATCC^®^ 29213™ was used as a quality control strain.

### 2.4. Identification of MRSA

*In vitro* detection of MRSA strains were applied by cefoxitin (30 µg) disk diffusion test and oxacillin salt agar that compromised of Mueller-Hinton agar (MHA, Merck, Darmstadt, Germany) containing 6 µg/mL of oxacillin (Sigma, USA) supplemented with 4% NaCl following the Clinical and Laboratory Standards Institute (CLSI) instructions [12]. The plates were inoculated with *S. aureus* isolates at a concentration of 1.5 × 10^8^ CFU/mL equal to the 0.5 McFarland tube and incubated at 35 °C for 16–18 h for cefoxitin (30 µg) disk diffusion and 24 h for oxacillin salt agar, respectively. In the cefoxitin (30 µg) disk diffusion, the isolates were considered MRSA if the inhibition zone around the disks was recorded ≤ 21 mm [12]. In the oxacillin salt agar, the existence of > 1 colony or light film of growth was considered as MRSA [12]. *S. aureus* ATCC^®^ 29213™ and *S. aureus* ATCC^®^ 43300™ were used as negative and positive quality control strains, respectively.

### 2.5. Antibiotic susceptibility testing of MRSA isolates

Antibiotic susceptibility testing of MRSA isolates were investigated by disk diffusion test on MHA medium according to the CLSI instructions [12]. The following antibiotic disks (Mast, UK) were used: azithromycin (15 µg), norfloxacin (10 µg), erythromycin (15 µg), rifampin (5 µg), chloramphenicol (30 µg), tetracycline (30 µg), and clindamycin (2 µg).

### 2.6. Preparation of starch-based ZnO-NPs

The previously described method was used to prepare the ZnO-NPs with minor modifications [13]. We examined different parameters to obtain an optimum synthesized ZnO-NPs. ZnO-NPs were prepared by the wet chemical precipitation method using the zinc nitrate 6-hydrate (Zn(NO_3_)_2_.6H_2_O) (Sigma-Aldrich, USA) in three concentrations (0.1 M, 0.05 M, 0.02 M) and sodium hydroxide (NaOH) (Sigma-Aldrich, USA) in concentrations of 0.2 M, 0.1 M, and 0.04 M as a precipitating agent in ratio 2:1. Also, in this method the soluble starch (Sigma-Aldrich, USA) was used as a stabilizing agent in concentrations (0.5%, 0.25%, 0.1%) for each prepared concentration of precursors mention above. The different concentrations of the primary precursors and stabilizer were prepared with deionized water and stirred vigorously using a magnetic stirrer till complete dissolution. The zinc nitrate solutions were kept under constant stirring at room temperature using a magnetic stirrer for 1 hour. Next, the starch solution was added and the mixture was magnetically stirred to obtain a homogeneous solution. Then, the NaOH solution was slowly added drop by drop at room temperature under vigorous stirring, which resulted in the formation of a white precipitate of the nanoparticles. The solution was allowed to settle overnight. Then, the precipitate was separated by centrifugation (10000 g for 10 min). The produced nanoparticles were washed three times with distilled water to remove the byproducts and the excessive starch particles that were bound to the formed nanoparticles. Finally, the nanoparticles were dried at 60–80 °C for overnight [13].

### 2.7. Characterization of starch-based ZnO-NPs

#### 2.7.1. Ultraviolet–visible (UV-Vis) spectroscopy analysis

The presence of nanoparticles was proved by UV-Vis analysis using the Shimadzu UV-1800 UV/Visible Scanning Spectrophotometer (Shimadzu, Kyoto, Japan) in the Department of Physics, University of Basrah. This device detected the surface plasmon resonance (SPR) peak of the prepared starch-based ZnO-NPs in the scanning range of 200–800 nm. An absorbance test was conducted on 1 cm quartz cells using starch-based ZnO-NPs dispersed in deionized water [13].

#### 2.7.2. X-ray diffraction (XRD)

Crystalline structure, nature of the phase, lattice parameters, and crystalline grain size of the starch-based ZnO-NPs were evaluated using XRD type Xpert MPD (Empyrean, Malvern Panalytical, Malvern, United Kingdom) in the Department of Physics, University of Basrah. The parameters were as follows: Cu-K 1 radiation (λ = 1.5406 Å) at 40 kV and 40 mA to work in Bragg–Brentano geometry with 2θ = (20–80)°, a speed of 2 sec/step and 0.02° step, and extract analysis 2θ = (0–80)° [14].

#### 2.7.3. Field emission scanning electron microscopy (FE-SEM) and energy dispersive X-ray spectroscopy (EDS)

The surface morphology and structure (mean particle size) of starch-based ZnO-NPs were evaluated using the FEI Nova NanoSEM 450 (FEI, Hillsboro, OR, USA) equipped with energy dispersive X-ray spectroscopy (EDS) in the Department of Physics University of Basrah. Starch-based ZnO-NPs were mixed with acetone and small drops of each sample were placed on a glass slide and allowed to dry. The samples were coated with thin layers of platinum to be conductive. The device was operated at a vacuum of the order 5–10 Torr. The acceleration voltage of the device was kept in the range of 10–20 kV. In the next step, the compositional analysis of the samples was carried out by EDS attached to the FE-SEM device. EDS analysis was used to determine the elemental compositions of the synthesized ZnO-NPs.

#### 2.7.4. Transmission electron microscopy (TEM)

The prepared solutions of starch-based ZnO-NPs in distilled water (Milli-Q®, Millipore Corporation, Bedford, MA, USA) were placed on carbon-coated copper grid and allowed to dry under ambient conditions. The particle size and the shape of starch-based ZnO-NPs were observed by a TEM microscope (Tecnai G2 200 kV TEM, FEI Electron Optics) with an accelerating voltage of 200 kV [15].

### 2.8. In vitro antibacterial assay of starch-based ZnO-NPs

Qualitative and quantitative assays were performed to evaluate the antibacterial effects of starch-based ZnO-NPs. The qualitative antibacterial effect of starch-based ZnO-NPs against clinical MRSA isolates was performed by standard disc diffusion method. The minimum inhibitory concentration (MIC) and minimum bactericidal concentration (MBC) were then determined using a broth microdilution assay as a quantitative assay.

#### 2.8.1. Disc diffusion method

For the disc diffusion method, the bacterial isolates were grown aerobically in nutrient broth for 24 hrs at 37 °C. Then, 100 µL of the bacterial suspensions (concentration equal to 2 × 10^8^ CFU/mL) was spread on sterile MHA plates. Sterile Whatman filter paper discs (5 mm) (Sigma-Aldrich, USA) saturated with 50 mg/L of prepared starch-based ZnO-NPs in distilled water (Milli-Q®, Millipore Corporation, Bedford, MA, USA) were placed on each inoculated plate. The cultured agar plates were incubated at 37 °C for 24 h. Finally, the zones of inhibition were recorded. Distilled water was used as the negative control [15]. The experiments were performed in triplicate. Results were estimated as mean ± the standard deviations (SD) of three replicates.

#### 2.8.2. Broth microdilution assay

MIC and MBC were determined for the concentration that showed the highest antibacterial effects with the disc diffusion method. About, 100 µL of starch-based ZnO-NPs was added into a sterile 96 well microtiter plate containing 100 µL of Mueller-Hinton broth (MHB, Merck, Darmstadt, Germany) to reach serially diluted concentrations of 200 to 0.2 µg/mL. Then, 100 µL of the bacterial suspensions (concentration equal to 2 × 10^6^ CFU/mL) was inoculated in each well to reach the concentration of 2 × 10^5^ CFU/mL. The microplate was incubated at 37 °C for 24 h. The MIC was described as the smallest amount of starch-based ZnO-NPs that prevented MRSA growth. Re-culturing (10 µL) of wells with no visible growth was performed on the MHA medium to determine the MBC. Incubation of the MHA plates was conducted aerobically at 37 °C for 24 h. The MBC for the examined strains was based on the starch-based ZnO-NP concentration at which bacterial growth was not detected. MHB inoculated with MRSA suspension and MHB alone were used as positive and negative controls, respectively. These experiments were repeated three times and the best observation was recorded as the final result [16],[17]. An MIC_90_/MBC_90_ was defined as a MIC/MBC that inhibits/kills 90% of MRSA isolates, while the MIC_50_/MBC_50_ was the MIC/MBC value that inhibits/kills 50% of isolates [18].

### 2.9. Data analysis

Statistical analysis of the data was performed using GraphPad Prism 9 (GraphPad Software, USA) and repeated measures ANOVA test. Data were presented as mean ± standard deviation (SD). The significant differences were considered based on the *P*-value < 0.05.

## 3. Results

### 3.1. *S. aureus* and MRSA isolates

In this study, a total of 61 (40.7%) *S. aureus* were isolated and identified from 150 clinical samples during the survey period. The isolates that showed round Gram-positive cocci with aggregate in clusters (irregular grapes) phenotype, positive catalase test, positive slide or tube coagulase test, fermentation of mannitol on MSA medium (changing the color of MSA from pink to yellow), and beta hemolysis on SBA were selected as *S. aureus* isolates. The results of cefoxitin (30 µg) disk diffusion and oxacillin salt agar showed that all 61 isolates were resistant to methicillin and were considered as MRSA. The most prevalence of MRSA isolates was found in ear pus samples (32.8%, 20/61), followed by sputum (29.5%, 18/61), urine (18.0, 11/61), nasopharynx (9.8%, 6/61), throat (6.6%, 4/61), and wound samples (3.3%, 2/61) (Table 1).

**Table 1.** Prevalence of methicillin-resistant *Staphylococcus aureus* (MRSA) isolates based on the clinical specimens.

Type of clinical specimens	Methicillin-resistant/Number	*Staphylococcus aureus* isolates/%
Wound	2	3.3
Sputum	18	29.5
Throat	4	6.6
Nasopharynx	6	9.8
Ear pus	20	32.8
Urine	11	18.0
Total	61	100.0

### 3.2. Antibiotic resistance patterns of MRSA isolates

The antibiotic resistance patterns of the MRSA isolates were shown in the Table 2. Accordingly, the most and the less resistance rates were against rifampin (60.7%) and chloramphenicol (11.5%), respectively.

**Table 2.** Antibiotic susceptibility testing of 61 methicillin-resistant *Staphylococcus aureus* (MRSA) isolates.

Antibiotics	Resistance/ N (%)	Susceptible/ N (%)
Oxacillin (1 µg)	61 (100)	__
Cefoxitin (30 µg)	61 (100)	__
Clindamycin (2 µg)	12 (19.7)	49 (80.3)
Tetracycline (5 µg)	18 (29.5)	43 (70.5)
Chloramphenicol (30 µg)	7 (11.5)	54 (88.5)
Rifampin (5 µg)	37 (60.7)	24 (39.3)
Erythromycin (15 µg)	21 (34.4)	40 (65.6)
Norfloxacin (10 µg)	21 (34.4)	40 (65.6)
Azithromycin (15 µg)	29 (47.5)	32 (52.5)

### 3.3. Characterization of starch-based ZnO-NPs

**Figure 1.**
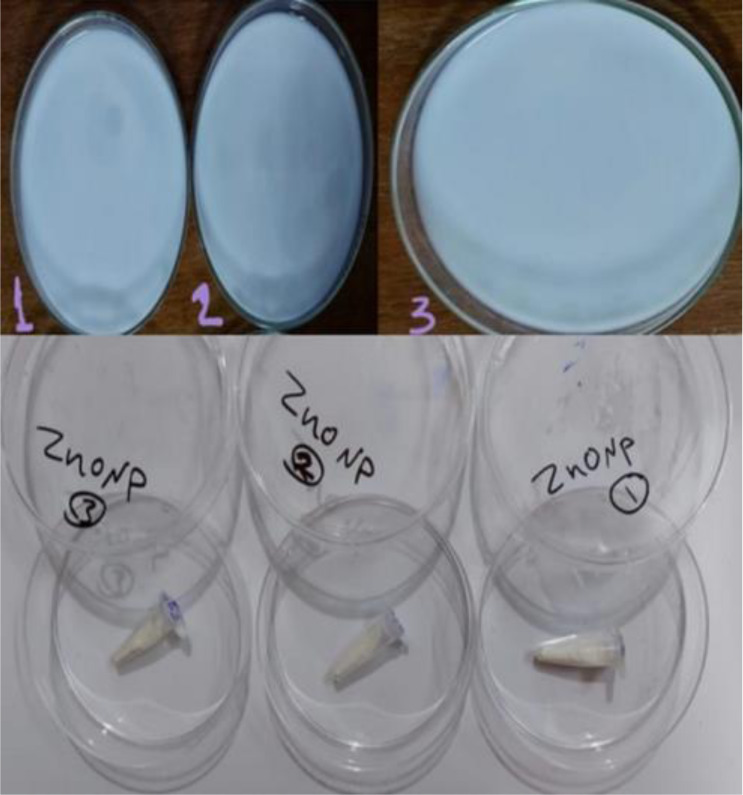
The pour and dried white powder of 3 different concentrations (1: 0.1 M, 2: 0.05 M, 3: 0.02 M) of the synthesized zinc oxide nanoparticles (ZnO-NPs).

The pour and dried white powder of 3 different concentrations (0.1 M, 0.05 M, 0.02 M) of the starch-based ZnO-NPs is shown in Figure 1.

#### 3.3.1. UV-Vis analysis

**Figure 2.**
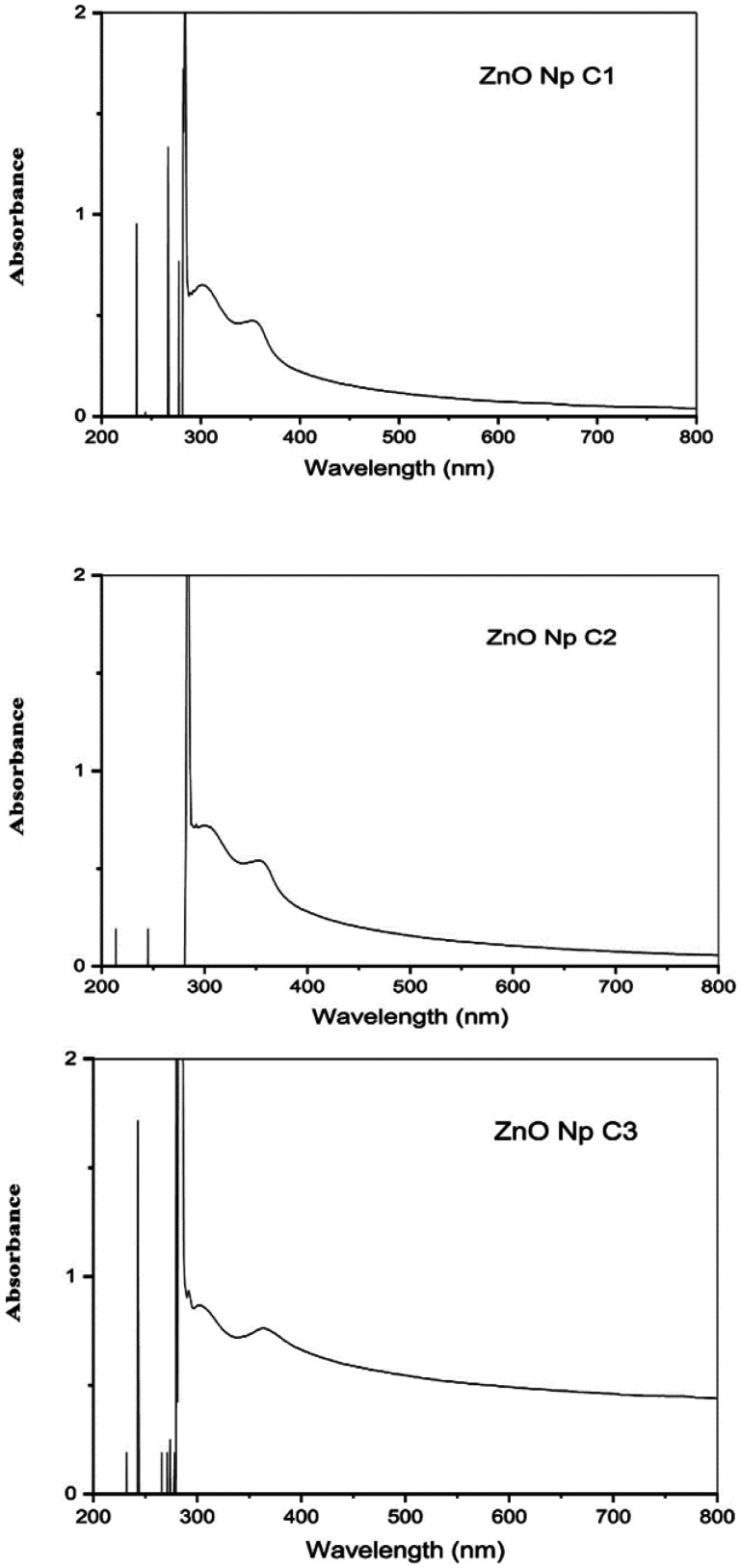
Ultraviolet–visible (UV-Vis) spectroscopy analysis of the three different concentrations (C1: 0.1 M, C2: 0.05 M, C3: 0.02 M) of the zinc oxide nanoparticles (ZnO-NPs) that prepared with 0.5%, 0.25% and 0.1% of soluble starch, respectively.

The UV-Vis spectra of the different three concentrations of starch-based ZnO-NPs (0.1 M, 0.05 M, 0.02 M) that prepared with 0.5%, 0.25% and 0.1% of soluble starch were shown in Figure 2. The three different concentrations of starch-based ZnO-NPs exhibited a strong absorption band in the region below 400 nm (at 360 nm) that was the characteristic for the ZnO-NPs (Figure 2).

#### 3.3.2. XRD analysis

**Figure 3.**
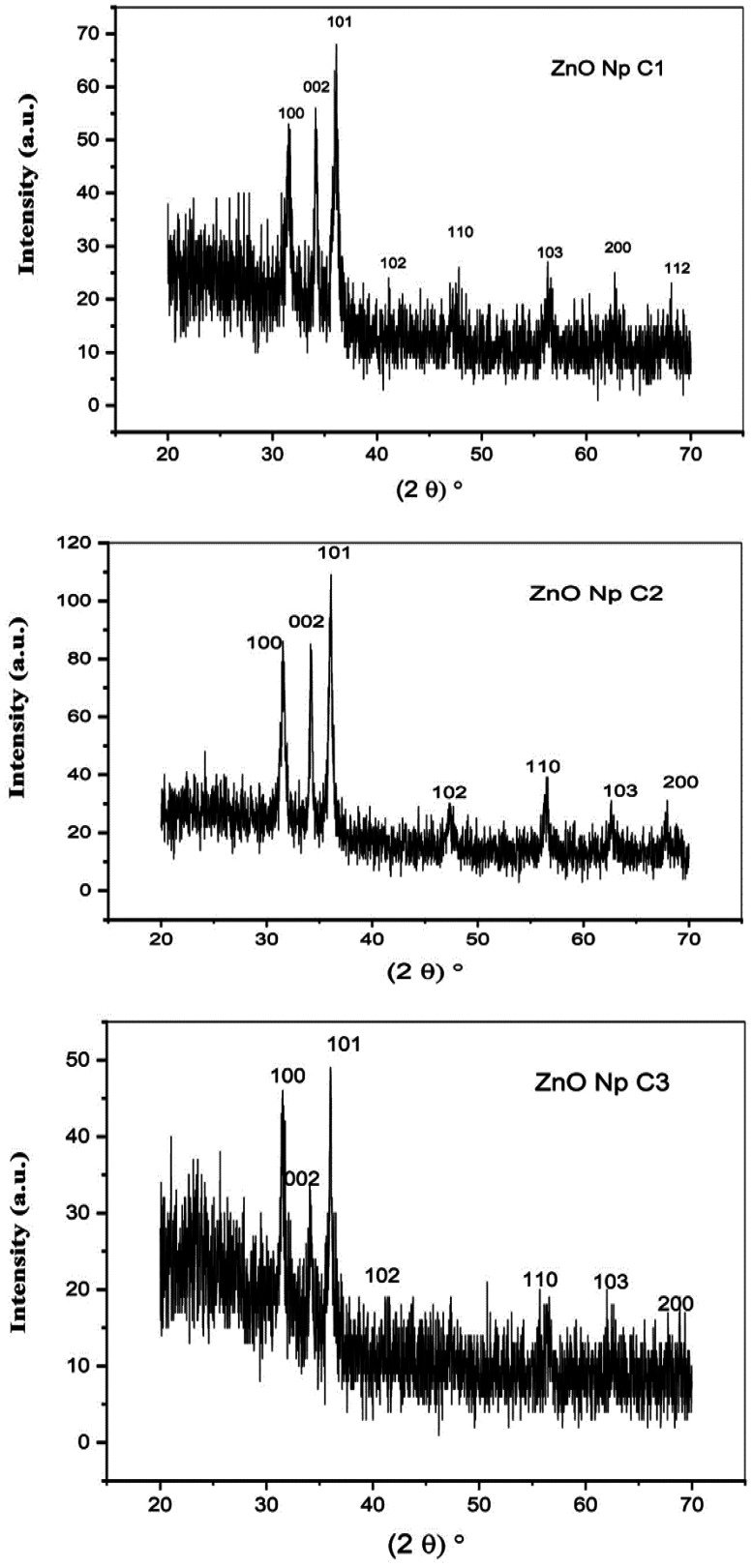
X-ray diffraction (XRD) of the three different concentrations (C1: 0.1 M, C2: 0.05 M, C3: 0.02 M) of the zinc oxide nanoparticles (ZnO-NPs) that prepared with 0.5%, 0.25% and 0.1% of soluble starch, respectively.

XRD patterns of the three concentrations of starch-based ZnO-NPs were shown in Figure 3. All diffraction peaks were obtained at 2θ values of 31.7°, 34.4°, 36.2°, 47.5°, 56.6°, 62.8°, 66.3°, 67.9° and 72.5° corresponding to (100), (002), (101), (102), (110), (103), (200) and (112) orientation planes, confirming the representative hexagonal wurtzite phase of the ZnO-NPs. The XRD spectra did not exhibit additional peaks associated with impurities, suggesting the high purity of the starch-based ZnO-NPs. Also, as evident from Figure 3, the signal sharpness indicated the high crystallinity of the starch-based ZnO-NPs. There were no differences in XDR patterns of different concentrations of ZnO-NPs as all of them showed the diffraction peaks at 2θ values of 31.7°, 34.4°, 36.2°, 47.5°, 56.6°, 62.8°, 66.3°, 67.9° and 72.5°.

#### 3.3.3. FE-SEM and TEM analysis

**Figure 4.**
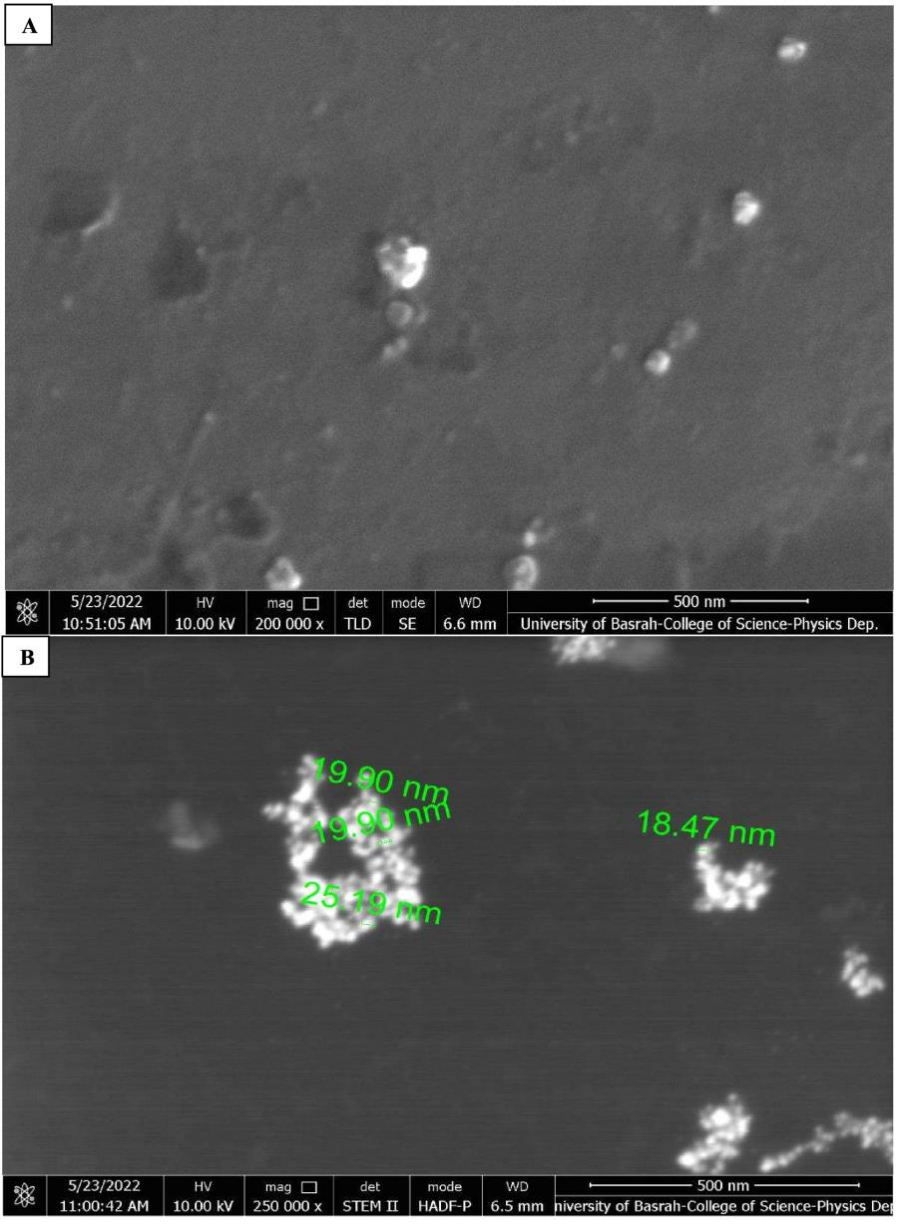
A: Field emission scanning electron microscopy (FE-SEM) morphology of the zinc oxide nanoparticles (ZnO-NPs). B: Transmission electron microscopy (TEM) morphology of the zinc oxide nanoparticles (ZnO-NPs).

The morphology of the starch-based ZnO-NPs was investigated by the FE-SEM and TEM as shown in Figure 4 A and B, respectively. The diameter of the ZnO-NPs was in the range of 18.47 to 25.19 (mean ± SD = 21.56 ± 3.09 and 22.87 ± 2.32 nm by FE-SEM and TEM, respectively). Starch-based ZnO-NPs displayed spherical morphology as shown by FE-SEM and TEM images. Also, a smooth surface was generally present on the particles, with uniform sizes and shapes. There were no significant differences (*P*-value > 0.05) in the mean ± SD of the size of three concentrations of the starch-based ZnO-NPs confirming the similarity of their size. Also, the shape of all synthesized ZnO-NPs showed spherical morphology confirming their shape similarity.

**Figure 5.**
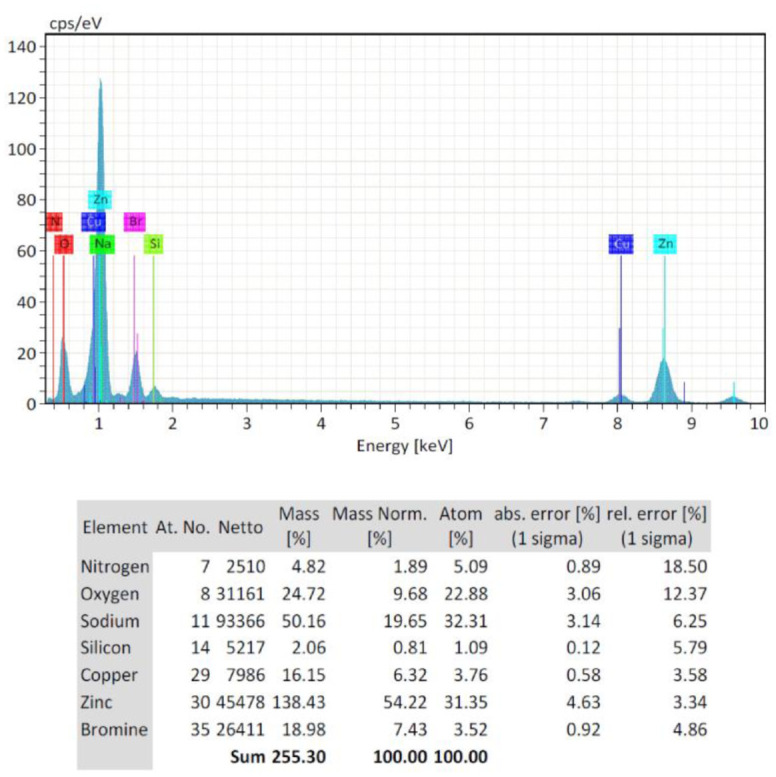
Energy dispersive X-ray spectroscopy (EDS) plot of the field emission scanning electron microscopy (FE-SEM) of C1 (0.1 M) starch-based ZnO-NPs.

**Figure 6.**
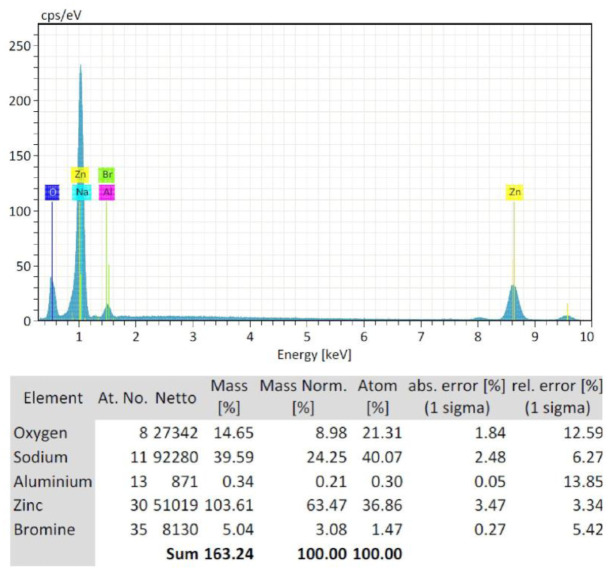
Energy dispersive X-ray spectroscopy (EDS) plot of the field emission scanning electron microscopy (FE-SEM) of C2 (0.05 M) starch-based ZnO-NPs.

**Figure 7.**
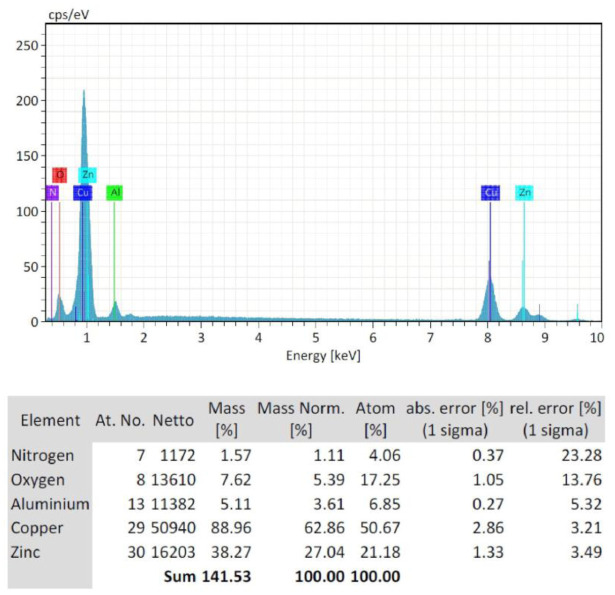
Energy dispersive X-ray spectroscopy (EDS) plot of the field emission scanning electron microscopy (FE-SEM) of C3 (0.02 M) starch-based ZnO-NPs.

EDS plots of the FE-SEM of three concentrations of starch-based ZnO-NPs were presented in Figures 5, 6, and 7. EDS analysis confirmed the presence of zinc (Zn) (54.22%) and oxygen (O) (9.68%) in C1 (0.1 M) concentration (Figure 5). Non-intentional dopants including Na (19.65%), Si (0.81%), Br (7.43%), Cu (6.32%), and N (1.89%) elements were also detected (Figure 5). This was probably due to the presence of substrate over which the ZnO-NPs samples were held for analysis. Also the zinc (Zn) (63.47%) and oxygen (O) (8.98%) were found in C2 (0.05 M) concentration (Figure 6). Non-intentional dopants including Na (24.25%), Alu (0.21%), and Br (3.08%) elements were also detected (Figure 6). Meanwhile, the zinc (Zn) (27.4%) and oxygen (O) (5.39%) were found in C3 (0.02 M) concentration (Figure 7). Non-intentional dopants including Cu (62.86%) as the major element, Alu (3.61%), and N (1.11%) elements were also detected (Figure 7). The carbon was not detected in any synthesized nanoparticles because the precipitates were produced from the reaction to obtain ZnO-NPs nanoparticles were separated by centrifugation at 10000 g for 10 min. Then, the produced nanoparticles were washed three times with distilled water to remove the byproducts and starch particles that were bound to the formed nanoparticles because the starch used in this method was a stabilizing agent and when the reaction was complete, we exclude it from the formed nanoparticles.

### 3.4. In vitro antibacterial effects of the starch-based ZnO-NPs

The results of the disc diffusion method showed that all concentrations of the starch-based ZnO-NPs had inhibitory effects on MRSA isolates. The 0.1 M concentration had the highest antibacterial effects with the mean ± SD of the inhibition zone of 17.62 ± 2.65 mm followed by the 0.05 M concentration with an inhibition zone of 16.03 ± 2.24 mm and the 0.02 M concentration with an inhibition zone of 12.7 ± 2.57 mm (Table 3). The MIC of the 0.1 M concentration was in the range of 25–50 µg/mL, while the MBC was in the range of 50–100 µg/mL. Also, the MIC90/MBC90 and the MIC50/MBC50 were 50/100 µg/mL and 25/50 µg/mL, respectively (Table 3).

**Table 3.** Antibacterial effects of the synthesized ZnO-NPs against methicillin-resistant *Staphylococcus aureus* (MRSA) isolates.

Antibacterial effects	Concentrations of ZnO-NPs		
	C1 (0.1 Mol)	C2 (0.05 Mol)	C3 (0.02 Mol.)
Inhibition zones of synthesized ZnO-NPs (Mean ± SD) (mm)	2.65 ± 17.62	16.03 ± 2.24	12.7 ± 2.57
MIC of 0.1 M concentration	25–50 µg/mL	
MBC of 0.1 M concentration	50–100 µg/mL	
MIC_90_/MBC_90_ of 0.1 M concentration	50/100 µg/mL	
MIC_50_/MBC_50_ of 0.1 M concentration	25/50 µg/mL	

MIC: minimum inhibitory concentration; MBC: minimum bactericidal concentration.

**Figure 8.**
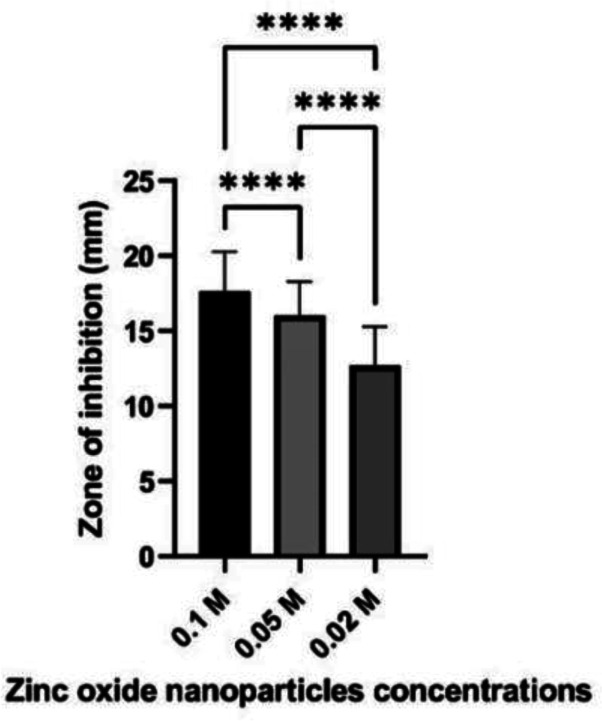
Inhibition zones of different concentrations (0.1 M, 0.05 M, 0.02 M) of zinc oxide nanoparticles against methicillin resistance *Staphylococcus aureus* isolates. The statistically significant differences were according to repeated measures ANOVA test (*P*-value < 0.05), **** = *P* < 0.0001.

The 0.1 M and 0.05 M concentrations of the starch-based ZnO-NPs showed significantly greater inhibition zones against MRSA isolates compared to 0.02 M concentration (*P*-value = 0.0001). Likewise, the inhibition zones of the 0.1 M concentration were significantly greater than those of 0.05 M ZnO-NPs (Figure 8).

## 4. Discussion

Various microbes have been prevented from growing on humans due to the use of zinc salt for decades. Also, there are extensive studies demonstrating the effectiveness of ZnO-NPs against pathogenic bacteria including *E. coli* and *S. aureus* [5],[8],[19]. However, the antibacterial effects of starch-based ZnO-NPs on MRSA isolates from Iraq is lacking. In this study, 61 MRSA isolates were collected from 150 different clinical samples of Iraqi patients, confirming the prevalence of 40.7%. This prevalence rate of MRSA was lower than previous reports from Iran (78.9%) [11] and Iraq (53.1%) [20], and was higher than studies from Italy (1.1%) [21] and Ghana (17.1%) [22]. Differences in prevalence rates may be explained by the differences in bacteria detection methods, examined populations, and studied sample types and sizes in various countries. The MRSA isolates showed relatively high resistance rates against azithromycin and rifampin (more than 40.0%), while the other antibiotics including chloramphenicol, tetracycline, clindamycin, norfloxacin, and erythromycin were more effective with resistance rates below 35.0%. In comparison to this study, previous research from Iran [11], found a higher resistance rate to azithromycin (100%) and erythromycin (98.3%) among MRSA isolates. However, in a previous study from Fiji [23], MRSA isolates showed a significantly lower resistance rate against clindamycin (0.0%), rifampicin (0.0%), and tetracycline (12.0%) that was in contrast to this study. These differences may be explained by the variations in the patients' demographics and geographical location that influence the resistance rates.

In this study, the qualitative and quantitative antibacterial assays showed promising effects of all synthesized starch-based ZnO-NPs against all MRSA isolates. These observations were in line with the previous studies from Egypt [5], Iraq [8], and Iran [16], in which the strong inhibitory effects of ZnO-NPs were found on multidrug-resistant *S. aureus*. The highest rate of inhibition was found at 0.1 M concentration with the mean ± SD of the inhibition zone of 17.62 ± 2.65 mm followed by the 0.05 M concentration (16.03 ± 2.24 mm) and the 0.02 M concentration (12.7 ± 2.57 mm). In previous studies, ZnO-NPs showed inhibition zones of 73.95 ± 2.17% at 10 mg/mL against vancomycin-resistant *S. aureus* (VRSA) and 16–21 mm against various Gram-negative and Gram-positive bacteria [8],[17]. In another study by Kamarajan et al. [24] from India, ZnO-NPs at a concentration of 10 µg/mL showed inhibitory effects against *Escherichia coli* (25 mm), *Pseudomonas aeruginosa* (23 mm), *S. aureus* (22 mm), and *Bacillus subtilis* (21 mm). The discrepancies in the inhibitory zone size in different studies may be due to the bacteria studied, shape, size, concentrations of the synthesized ZnO-NPs, and method used to synthesize ZnO-NPs.

Previous studies have found that the shape, size, concentrations of the synthesized ZnO-NPs, and the method to synthesize ZnO-NPs affect the antibacterial properties of the nanoparticles [24]–[27]. In this study, the highest concentrations of the starch-based ZnO-NPs exhibited significantly greater inhibition zones compared to the lowest concentrations. These results were consistent with the previous studies in which higher concentrations of ZnO-NPs showed stronger antimicrobial effects [8],[25],[26]. However, some studies showed that the inhibition zone of nanoparticles starts to shrink beyond an optimal concentration [28],[29]. One of the possible reasons may be due to the accumulation of nanoparticles in high concentrations and the inability to penetrate into bacterial cells [28],[29].

ZnO-NPs are believed to act in four distinct ways including releasing Zn^2+^ ions, damaging the cell wall, producing reactive oxygen species (ROS), and by ZnO-NPs internalizing [25]. The antibacterial activity of ZnO-NPs depends on their penetration into bacterial cells. Thus, the antibacterial effects of ZnO-NPs can be evaluated by the broth dilution method as a precise and confirmative assay [5]. In this study, the broth microdilution assay revealed the MIC of the starch-based ZnO-NPs in the range of 25 to 50 µg/mL at the 0.1 M concentration. Also, the MBC was in the range of 50 to 100 µg/mL. These values were lower than a previous study that reported ZnO-NP MICs ranging from 128 to 2048 µg/mL against *S. aureus* isolates [5]. MIC values in this study were also lower than those reported by Jasim et al. [8] against VRSA isolates (625 µg/mL). However, in a previous study by Tănase et al. [14] from Romania, the chemical and *Saponaria officinalis* extract-mediated ZnO-NPs showed lower MICs (<20 µg/mL) against standard strains of *S. aureus*, *P. aeruginosa*, *E. coli*, and *Candida albicans*. The differences among studies may be due to the used methodology, the antibiotic resistance patterns of examined bacteria, and the structural nature of the synthesized ZnO-NPs.

In this study, the structural nature of the starch-based ZnO-NPs were investigated by various methods. The UV-Vis analysis showed that three concentrations of starch-based ZnO-NPs (0.1 M, 0.05 M, 0.02 M) exhibited a strong absorption band at 360 nm which was characteristic of the ZnO-NPs. This observation was in good parallel with the previous studies from Egypt [13], Romania [14], and Jordan [30] which showed the absorption peaks of ZnO-NPs below 400 nm. The shape, size, and method of fabrication of ZnO-NPs are all factors influencing the absorption peak. In general, ZnO-NPs exhibit a UV-Vis spectroscopic peak between 350 and 390 nm [30]. Moreover, the XRD analysis of the three concentrations of the starch-based ZnO-NPs confirmed the representative hexagonal wurtzite phase, high purity, and high crystallinity of the starch-based ZnO-NPs. These results were consistent with the previous observations from Malaysia [15] and Jordan [30]. Another observation of this study was the spherical morphology of the starch-based ZnO-NPs with a diameter of 21.56 ± 3.42 and 22.87 ± 3.91 nm by FE-SEM and TEM, respectively. There was no any significant difference among three concentrations of the starch-based ZnO-NPs in terms of size and shape. In contrast to this study, Saleemi et al. [15], showed rod-shaped morphology of standard ZnO-NPs with the diameter of 49.39 ± 22.54 nm [15]. Alshraiedeh et al. [30] reported spherical ZnO-NPs with a size of 100 nm in their study.

From a future perspective, it is recommended to examine the synergistic effects of the synthesized starch-based ZnO-NPs in combination with standard antibiotics or other chemical or plant-based materials against different pathogens and cancer cell lines. Previous studies have shown the significant effects of combining nanoparticles with other materials against microorganisms [31],[32]. Although several studies have investigated the antibacterial effects of ZnO-NPs against MRSA isolates, but in each of them, the different researchers looked forward to finding more effective nanoparticles in terms of their shape, quality, and antibacterial effects. The novelty of this study was the synthetization of relatively smaller nanoparticles in comparison to previous studies. Also, the synthesized nanoparticles showed promising antibacterial effects in low concentrations (0.02 Mol). However, this study had several limitations as follows: lack of investigation of starch-based ZnO-NPs against other Gram-positive and Gram-negative bacteria, lack of *in vivo* experiment, and lack of time-kill kinetics assay.

## 5. Conclusion

UV-Vis, XRD, FE-SEM, and TEM analysis showed the crystalline organization, spherical shape, and smooth surface of the starch-based ZnO-NPs with a size below 27 nm. Qualitative and quantitative antimicrobial assays showed the promising effects of the starch-based ZnO-NPs against clinical MRSA isolates with MIC ranging from 25–50 µg/mL. Further *in vivo* experiment is needed to reveal the mechanism of action of the synthesized starch-based ZnO-NPs.
